# Experiment and Optimization for Simultaneous Carbonation of Ca^2+^ and Mg^2+^ in A Two-phase System of Insoluble Diisobutylamine and Aqueous Solution

**DOI:** 10.1038/srep10862

**Published:** 2015-06-03

**Authors:** Wenlong Wang, Man Wang, Xin Liu, Peng Wang, Zhenqian Xi

**Affiliations:** 1 National Engineering Laboratory for Coal-fired Pollutants Emission Reduction, Shandong University, Jinan 250061, China; 2 Department of Chemical and Environmental Engineering, The University of Nottingham, University Park, Nottingham NG7 2RD, UK

## Abstract

An optimized approach of CO_2_ fixation in Ca^2+^/Mg^2+^-rich aqueous solutions using insoluble amine as an enhancing medium was reported. Apparent basicity was verified to be an effective indicator for the selection and optimization of organic amine systems and finally the diisobutylamine + n-octanol system was selected to enhance the carbonation reactions of CO_2_ in an artificial Ca^2+^/Mg^2+^-rich solution. In our experiments, when the volume ratio of insoluble organic phase to aqueous one was 2:1 and the reaction temperature was 28 °C, 92% of Ca^2+^ and 80% of Mg^2+^ could be converted to calcium and magnesium carbonate precipitates within 5 min of reaction with the bubbling-in of CO_2_. The organic amine system could be regenerated by using carbide slag as the regeneration agent and could still show attractive enhancement performances after 7 rounds of carbonation-regeneration experiments. In this way, the CO_2_ capture and sequestration was realized within one single process, with value-added Ca/Mg carbonates being the byproducts. In view of the vast availability of Ca^2+^/Mg^2+^-rich aqueous solutions and the feasible technical coordination with desalination industry, this novel process may have a good application potential in the future.

Various scientific observations show the concentration of CO_2_ is rising continuously and was once observed to break through 400 ppm according to the Mauna Loa Observatory on 9^th^ May, 2013^1^. In addition to source reduction, carbon capture and storage (CCS) is also expected to play a significant role in CO_2_ reduction for climate change mitigation^2^,^3^,^4^,^5^,^6^,^7^,^8^. Further, CCUS (carbon capture, utilization and storage) is more preferentially used by scientists because it covers wider and more attractive ideas in this domain^9^,^10^,^11^,^12^.

Our working group has all along been devoting to the development of a novel CCUS conception, i.e. trying to use Ca^2+^/Mg^2+^-rich aqueous solutions, such as concentrated seawater, subsurface brine and industrial effluents, to fix CO_2_ through enhancing the precipitation of carbonates. In this way, not only may CO_2_ be directly transformed into carbonate precipitates and permanently stored as a value-added byproduct^13^,^14^,^15^, but also the precipitation of most Ca and Mg ions endows the corresponding aqueous solutions a much better condition for post-treatment like desalination^16^,^17^, where the water production ratio can be dramatically increased by reducing scaling caused by carbonation precipitation. Our previous work first demonstrated the feasibility and potential of CO_2_ fixation through thermodynamic analyses and kinetic experiments and confirmed that over 90% of the Ca^2+^ and Mg^2+^ in seawater could be converted into carbonates on temporal scales of engineering when the pH level of reaction system could be raised steadily by employing something as a pH regulator^18^. Subsequently, the seeking for proper pH regulator was focused on, and eventually, insoluble amine, such as tributylamine, was confirmed to be an efficient candidate^19^.

In our built-up process, when alkaline amine extractants are added into a Ca^2+^/Mg^2+^-rich aqueous system, the equilibrium of CO_2_ dissolution in water will be broken. As shown in Eq. (1), NR_3_ can extract H^+^ from water phase into organic phase to form NR_3_∙H^+^. The loss of H^+^ raises the pH of the water phase and triggers the rise of CO_3_^2−^ concentration in the case of sufficient CO_2_ partial pressure following Eq. (2). Accordingly, CaCO_3_ and⁄ or MgCO_3_ are generated as in Eq. (3). After precipitation, the organic phase of amine, owing to its insolubility, can spontaneously get apart from the aqueous solution to form two liquid phases. Meanwhile, high-quality carbonate byproducts are obtained, and the amine phase can be regenerated via some alkaline minerals like carbide slag or aqueous ammonia.


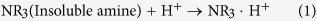



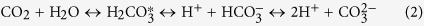



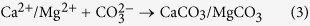


In particular, the whole process enables operation at a room temperature and there is no additional energy consumption except for the necessary mechanical drive. It means that the capture of CO_2_ and its storage as value-added byproducts are realized in just one ordinary-temperature procedure.

However, when tributylamine was used as the pH regulator and n-butyl alcohol as its diluent in our previous work, the precipitation degree of Mg^2+^ was very limited in spite of a good precipitation of Ca^2+^, which was mainly due to the insufficient alkalinity enhancing ability of the amine-diluent combination. It told us that the organic amine system needed optimization in view of the tremendous diversity of combination of insoluble amines and diluents. In this paper, apparent basicity^20^ was proved to be effective and was adopted as an indicator for the selection and optimization of organic amine systems, and diisobutylamine plus n-octanol was finally selected. Then, the complete CO_2_ fixation experiments were carried out with artificial Ca^2+^/Mg^2+^-rich solution in a self-designed continuous experimental setup. The carbonation dynamics of Ca^2+^and Mg^2+^, the influence of some factors, as well as the regeneration of diisobutylamine, were all investigated. An overall profile of the CO_2_ fixation through enhancing carbonation precipitation in Ca^2+^/Mg^2+^-rich aqueous solutions was reported.

## Methodology

### Materials

Ca^2+^/Mg^2+^-rich aqueous solutions are widespread and abundant on the earth. For instance, concentrated seawater as a typical one is being increasingly produced by desalination industries as waste in recent years. Its concentrations of Ca^2+^ and Mg^2+^ may be twice those of natural seawater, amounting to 0.82 g/L and 2.58 g/L respectively^17^. Concentrated seawater is a possible candidate to capture CO_2_ through enhancing carbonate precipitation with tremendous application potentials. Because the ions like Na^+^, K^+^ and SO_4_^2-^ in seawater were proved not to interfere with the mineralization process in our previous work^13^, in this paper we only took MgCl_2_ and CaCl_2_ to prepare a Ca^2+^ /Mg^2+^-rich aqueous solution instead of the practical concentrated seawater. The concentrations of MgCl_2_ and CaCl_2_ were taken 10.21 g/L and 2.28 g/L respectively. All the reagents used, including the diisobutylamine, n-octonal, MgCl_2_ and CaCl_2_, were analytical pure purchased from Sinopharm, China.

### Experimental setup

**Figure 1:**
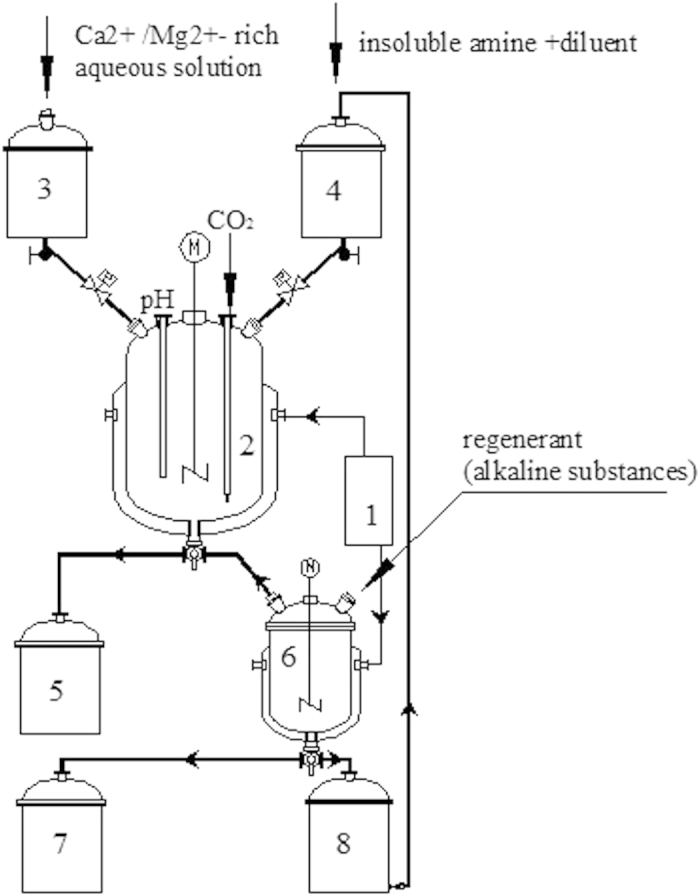
Experimental setup. 1. Thermostatic circulator 2. Carbonation reactor 3. Aqueous phase accumulator 4. Organic phase accumulator 5. Aqueous phase collector after carbonation 6. Regeneration reactor 7. Collector of the residue materials 8. Organic phase collector after regeneration.

A set of glass reaction system was specially designed for the CO_2_ fixation process and shown in Fig. 1. Therein, the carbonation reactor and regeneration reactor are the two main parts to carry out the mineralization of CO_2_ and the regeneration of insoluble amine respectively. The inlet of regeneration reactor is connected to the outlet of carbonation reactor, and the regenerated organic amine phase is guided back to the carbonation reactor using a circulating pump. The 1L carbonation reactor has a jacketed glass structure, through which the reaction temperature can be controlled via circulating water provided by a thermostat. And, a stirrer is equipped in the carbonation reactor to keep the sufficient blending of the organic and aqueous phases. All the rest accumulators and collectors are 500 mL instruments with lids. The whole process of CO_2_ mineralization and amine regeneration can be conducted consecutively in this setup.

### Experiment 1 - Selection of insoluble amine system

Since the concentration of Mg^2+^ is twice higher than that of Ca^2+^ in seawater-based solution, it was a big defect for our previous tributylamine + n-butyl alcohol system that was only able to enhance the precipitation of the later ions. Since there are thousands of different combinations of amine+ alcohol systems for selection, it is possible for us to find a better system which can overcome the limitations in our previous work. However, to carry out the optimization, it would be inefficient and costly to screen the combinations one by one following the way of mineralization experiments in our previous papers. Developing a convenient method or finding a common parameter to reflect the mineralization ability of amine is in urgent need.

As shown in Eq. (1)-(3), the mineralization capacity of an amine-diluent system is decided by its extraction ability for H^+^ in essence. Apparent basicity, pK_a,B_, as a parameter defined by Eyal at al^20^, had ever been successfully used to describe the ability of varied amine-diluent systems to extract mineral acid. Thus, this parameter was referred to in our study. When the apparent basicities were measured, a certain concentration of amine in diluents and a 1/2 this concentration of HCl solution were mixed at the volume ratio of 1:1 in a 100 mL flask, i.e. the molar of HCl was kept 1/2 of that of amine in the mixed system; then, the mixed system was stirred for 120 min at the temperature of 25 ± 0.1 °C; after another 30 min of standing to let the aqueous phase and organic phase get stratified, the aqueous phase was separated to measure its pH value. The pH value obtained in these cases can be approximately considered as the apparent basicity of the amine + diluent system.

Based on the experimental data obtained when we used tributylamine + n-butyl alcohol previously, the reliability of employing apparent basicity to reflect the enhancement abilities of CO_2_ mineralization for our study was first tested. Then, different combinations of amine plus diluent were compared by measuring their apparent basicities under the same operation conditions.

**Figure 2:**
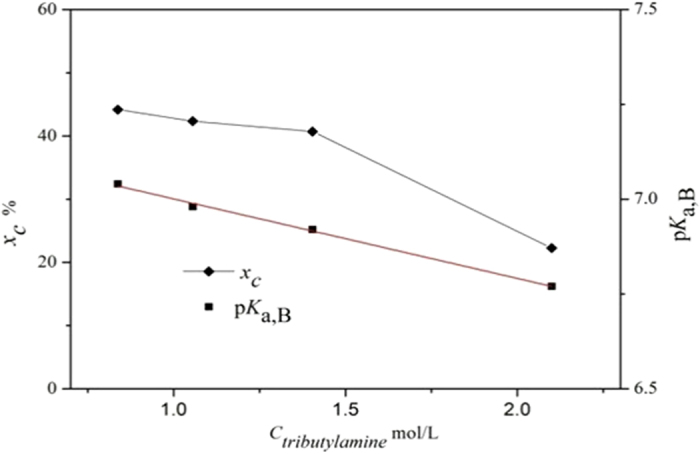
Relationship between apparent basicity and Ca^2+^ mineralization ratio with the change of tributylamine concentration in n-butyl alcohol.

Figure 2 illustrates the corresponding relationship between apparent basicity pK_a,B_ and Ca^2+^ mineralization ratio x_c_ when tributylamine + n-butyl alcohol system was employed to enhance CO_2_ fixation. It can be clearly seen the two parameters have the same trend when the concentration of tributylamine in n-butyl alcohol changes, which means apparent basicity can be used as an effective indicator to screen amines. Moreover, the lower concentration of tributylamine corresponding to higher pK_a,B_ and x_c_ indicates that diluents also play an important role in improving the abilities of tributylamine for H^+^ extraction and mineralization enhancement.

**Figure 3:**
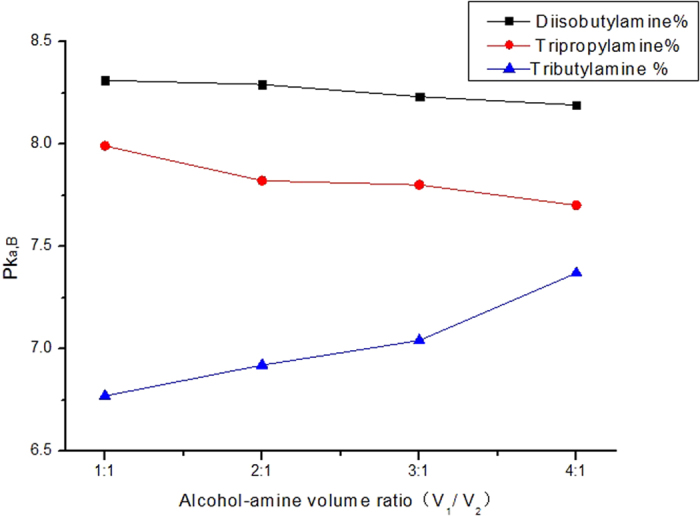
Apparent basicities of three different insoluble amine + diluent systems at different volume ratios (diisobutylamine + n-octanol, tripropylamine + n-octanol, tributylamine + n-butyl alcohol).

Figure 3 gives the apparent basicities when different combination systems of insoluble amine + diluent were built. Therein, diisobutylamine and tripropylamine have similar molecular configuration to tributylamine and n-octanol has lower solubility in water than n-butyl alcohol. It can be seen that both diisobutylamine + n-octonal and tripropylamine + n-octanol systems have higher apparent basicities than the system of tributylamine + n-butyl alcohol. In addition, the pK_a,B_ of the two new systems do not rise with the increase of diluent volume like tributylamine + n-butyl alcohol system, but decline slightly instead, which means their relationship is indefinite and might be influenced by the difference of octanol-water partition coefficient. In view of the prominent performance of diisobutylamine + n-octonal system, it was selected as the new mineralization enhancement system in our following experiments and a low dilution ratio of 1:1 was adopted for amine and n-octanol.

### Experiment 2 - Carbonation

During the carbonation experiments, the Ca^2+^/Mg^2+^-rich aqueous solution and the insoluble amine + diluent were first added into the carbonation reactor in a certain proportion. The two non-miscible organic and aqueous phases were then well mixed by the stirrer at a speed of 500 rpm. At this moment, a 0.3 L/min CO_2_ gas stream was guided into the reactor. The reactor was kept at 28 °C and the change of pH was monitored by a pH meter. With the CO_2_ being bubbled in, the pH of the liquid system declined gradually. When the pH fell to 8.60, the experiment would be terminated.

After a standing of several minutes, the mixed system became stratified steadily. Thereupon, the lower aqueous phase together with the precipitate was guided into collector 5 and the organic phase in the upper layer was guided into the regeneration collector 6. The precipitate was filtered and then dried at 110 °C for 24 h; XRD and SEM were adopted to analyze its properties. The concentrations of Ca^2+^ and Mg^2+^ in the aqueous phase were measured with the method of EDTA titration:


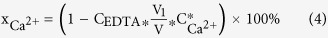



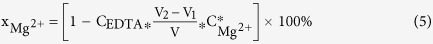


where, C_EDTA_ is the concentration of EDTA standard solution; V_1_ is the volume of EDTA standard solution consumed in the titration of Ca^2+^; V_2_ is the volume of EDTA standard solution consumed in the titration of Mg^2+^; V is the volume of subnatant liquid that was used in the titration experiment;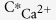 and 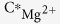 represent the concentrations of Ca^2+^ and Mg^2+^ in the initial artificial solution respectively.

### Experiment 3 - Regeneration

During the regeneration of insoluble amine in the organic phase, carbide slag was taken as the regeneration agent and added into the regeneration reactor at a molar ratio of 1.5:1 for carbide slag vs. acidified amine. The regeneration process lasted about 15 min under stirring at a speed of 500 rpm and 28 °C.The regeneration of diisobutylamine from the form of NR_3_⋅H^+^ to NR_3_ meant its recovery as the mineralization enhancement agent. The concentrations of H^+^ in the organic phase before and after regeneration could be titrated by NaOH (0.1 mol/L). Then the regeneration ratio of diisobutylamine can be expressed as:


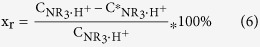



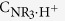
 and 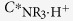 represent the concentrations of NR_3_⋅H^+^ before and after the regeneration, respectively.

## Results and Discussion

### Carbonation dynamics

**Figure 4:**
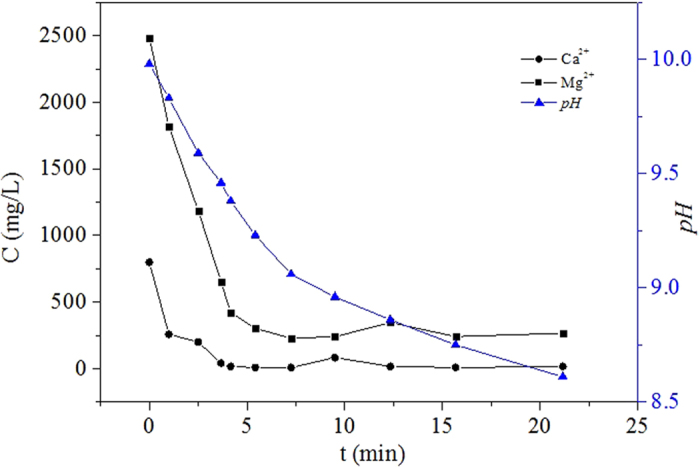
Changes of pH and concentration of Ca^2+^ and Mg^2+^ with reaction time in carbonation process.

In the typical carbonation experiments with diisobutylamine +n-octonal as the enhancement system, the organic phase and aqueous phase were mixed at the volume ratio of 2:1 and the reactions were carried out at 28 °C. Before CO_2_ was bubbled in, the mixed system was only slightly turbid and the pH value kept at about 10.0. With the continuous bubbling-in of CO_2_, the pH declined from 10.0 to 9.2 within 5 min and to 8.6 within 20 min. Meanwhile, the mixed system was observed to be getting more and more turbid. At some non-fixed intervals, the mixed system was sampled and the corresponding concentrations of Ca^2+^ and Mg^2+^ were measured. All the obtained results were shown in Fig. 4.

It can be clearly noticed in figure 4 that both the pH and the concentrations of Ca^2+^ and Mg^2+^ took a declining trend with the increase of reaction time. The concentration of Ca^2+^ dropped dramatically within 4 min and almost kept unchanged in the following period. There was a total drop of about 98% finally. The decline of the concentration of Mg^2+^ was not as sharp and quick as that of Ca^2+^ but it also had a drop of 91% within 5 min and changed unnoticeably later. It meant that most of the Ca^2+^ and Mg^2+^ could be precipitated in our experiments by using the diisobutylamine + n-octanol system as the pH regulator. The vast-majority precipitation of Mg^2+^ in this experiment, which was not obtained in our previous work while using tributylamine + n-butyl alcohol, indicated that the carbonation enhancement method here was more suitable for Mg^2+^-rich aqueous sources and had much better application potentials than before.

The different precipitation rate of Ca^2+^ and Mg^2+^ should be caused by their distinct solubility product constants, Ksp, as well as their different initial concentrations. The Ksp of MgCO_3_ is 1.15 × 10^−5^ at 298 K, while that of CaCO_3_ is 4.95 × 10^–9,^^13^. Because the latter is 4 orders of magnitude lower than the former, CaCO_3_ has a much higher tendency of precipitation forming and will be sure to precipitate in priority at a certain concentration of CO_3_^2−^.

### Effects of reaction conditions

**Figure 5:**
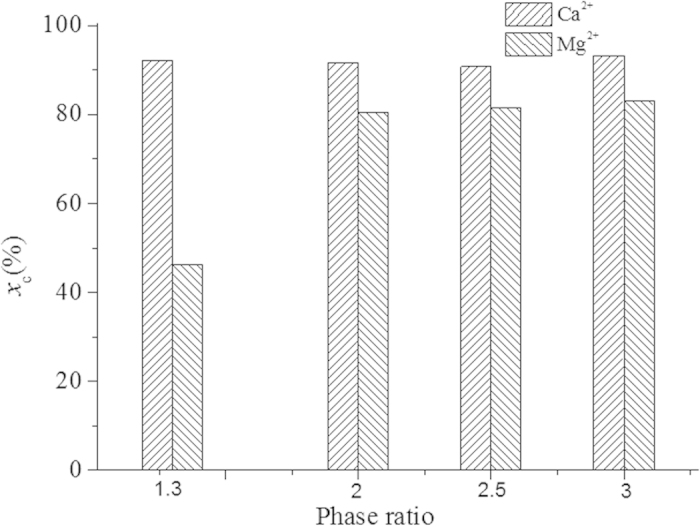
Effect of phase ratio on carbonation process.

The volume ratio of organic and aqueous phases, i.e. phase ratio, was proved to affect the carbonation performance greatly in our previous experiments^21^, so the effect of phase ratio was also investigated here. Figure 5 shows the percentages of precipitation of Ca^2+^ and Mg^2+^ at different phase ratios. When the phase ratio increased from 1.3:1 to 3:1, the precipitation percentage of Ca^2+^ did not vary much and all through remained at over 90%. However, the precipitation percentage of Mg^2+^ was only 46% at the phase ratio of 1.3:1 and yet increased to 80% at 2:1. While, there was very limited further growth, only from 80% to 83%, when the phase ratio changed from 2:1 to 3:1.The reason for this should be that the increasing phase ratio could enhance the extraction of H^+^ from the aqueous phase and thereupon provide more drives for the rise of CO_3_^2-^ concentration which triggered the precipitation of Mg^2+^. The less influence on Ca^2+^ precipitation should also be owing to the much smaller Ksp of CaCO_3_ than MgCO_3_. Since the effect of mineralization enhancement was not prominent at a phase ratio of higher than 2:1, the phase ratio can be fixed at 2:1 in practical processes.

**Figure 6:**
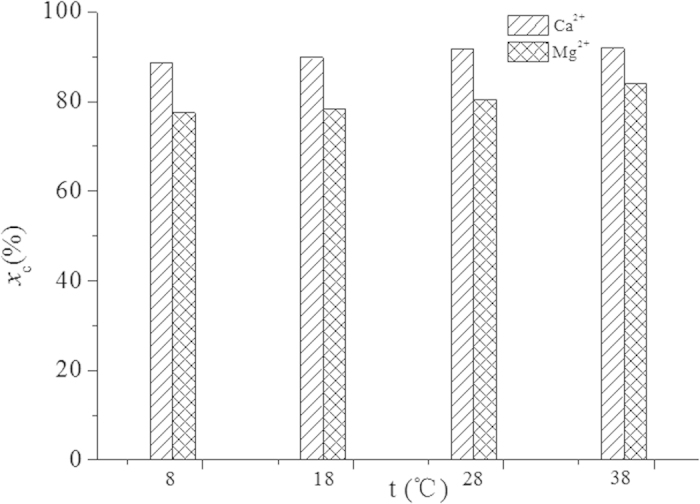
Effect of temperature on carbonation process.

The influence of temperature on the carbonation process was also tested. Figure 6 shows the precipitation changes of the Ca^2+^ and Mg^2+^ at a phase ratio of 2:1 under different temperatures. It can be found that, when the reaction temperature rose from 8 °C to 38 °C,the carbonation percentages of both Ca^2+^ and Mg^2+^ increased to some extent; yet, the increase for Ca^2+^ was very slight while that for Mg^2+^ was more noticeable, from 72% to 84%. It meant that the increase of temperature from 8 °C to 38 °C could be conducive to the carbonation reactions for the diisobutylamine + n-octanol system. In our previous work based on tributylamine + n-butyl alcohol system, however, the increase of temperature from 10 °C to 40 °C had sharply lowered the mineralization ratio of Ca^2+^. Accordingly, the diisobutylamine + n-octanol system has substantial advantages from multiple angles. The favorableness of higher temperature makes the process suitable to directly capture the CO_2_ in some exhaust gases like the flue gases from fossil-fuel power plants.

### Precipitate analysis

**Figure 7:**
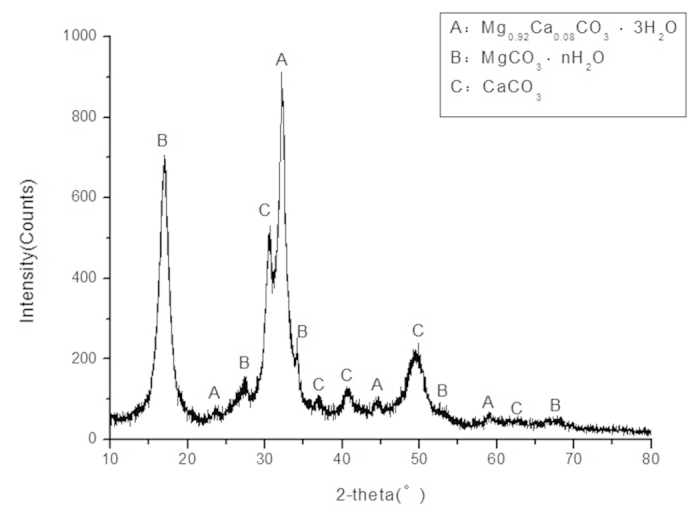
XRD diffractogram of the precipitate product.

For the obtained precipitate product, which took the form of white powder after filtering and drying, XRD and SEM were adopted to determine its mineral compositions and properties. Figure 7 shows its XRD diffractogram. Through careful analysis referring to standard XRD patterns, the precipitate product was determined to be the mixture of magnesium and calcium carbonates. The main crystalline forms include Mg_0.92_Ca_0.08_CO_3_·3H_2_O, MgCO_3_·nH_2_O and CaCO_3_. These results confirmed that the carbonation reactions entirely met our expectations. Thus, the diisobutylamine is an effective candidate in enhancing the precipitation of Ca^2+^ and Mg^2+^ in their specific aqueous solutions.

**Figure 8:**
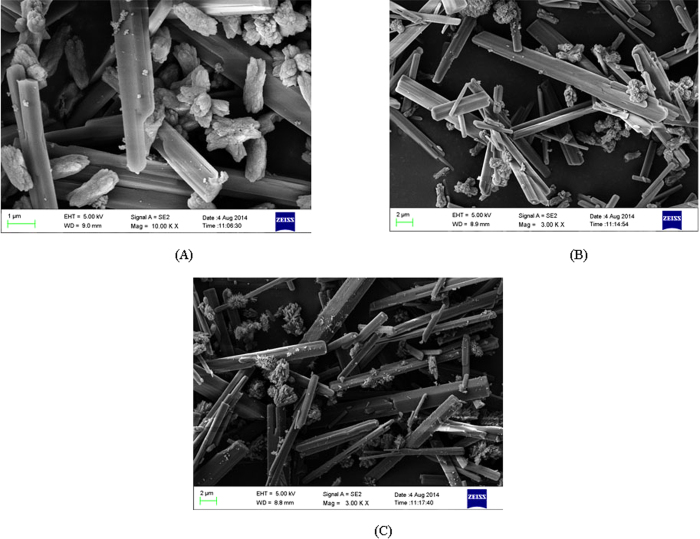
SEM morphologies of the precipitate product (A) obtained at 18° C carbonation (B) obtained at 8° C carbonation (C) obtained at 28° C carbonation by using the regenerated amine.

Figure 8 gives the SEM images of the precipitate product samples obtained (A)at 18 °C carbonation, (B)at 8 °C carbonation, and(C)at 28 °C carbonation. Therein, sample(C) was produced by using the regenerated amine as the enhancement agent. Two crystalline structures, in irregular shape or in rod shape, can be observed in the SEM images. The fraction in irregular shape might be the mixture of calcium carbonate and magnesium calcium carbonate, while the fraction of rod shape should be magnesium carbonate. Because the amount of Mg^2+^ were much more than that of Ca^2+^, it is reasonable that the rod-shaped MgCO_3_ is dominant. In addition, it can be found that the crystal forms are similar in the three SEM figures, which means that the change of reaction temperature and whether or not the amine experienced regeneration had little impact on the formation of carbonate precipitate.

In addition, since the carbonate products are in excellent state of particle sizes, color appearance and composition stability, they may have vast forms of application in industrial fields. For instance, there is even no need for further disposal when they are used as filling materials in paper, printing, rubber, polymer, etc.

### Regeneration of pH regulator

The regeneration performance of the pH regulator, namely the insoluble organic phase, determines its operation life and industrial application prospect. During the mineralization process, the insoluble diisobutylamine extracts hydrogen ions from the aqueous phase into the organic phase to form NH(C_4_H_9_)_2_·H^+^, so the regeneration of the amine should be a process of converting NH(C_4_H_9_)_2_·H^+^ back to NH(C_4_H_9_)_2_. The regeneration can be achieved by the addition of some basic substances, such as NaOH, Ca(OH)_2_, ammonia water, etc, or through directly heating. A kind of basic industrial waste, carbide slag, was used to regenerate NH(C_4_H_9_)_2_ here. The performances of the regenerated organic phase were tested in different experimental cycles of carbonation enhancement.

**Figure 9:**
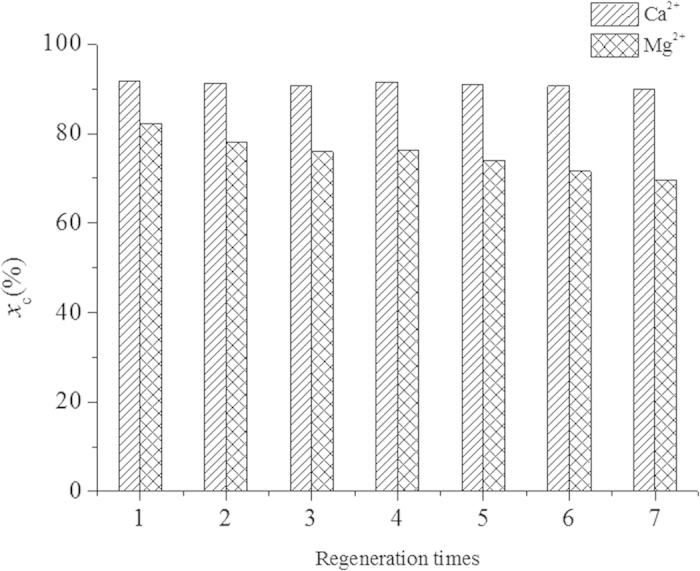
Carbonation performances of the diisobutylamine + n-octanol system in different cycles of carbonation enhancement.

The regeneration of diisobutylamine was carried out for 7 rounds of carbonation-regeneration experiments at the phase ratio of 2:1 and 18 °C. Figure 9 gives the carbonation performance of each round. It can be found that there were declines for the precipitation percentages of both Ca^2+^ and Mg^2+^. For Ca^2+^, the decline was slight, from 92% in the 1st round to 89% in the 7th round; while for Mg^2+^ the decline was from 82% to 69%. The reason for the declines might be attributed to the denaturation of a portion of diisobutylamine, which might have been converted to carbamate during the carbonation process and could not be regenerated by base any more. Nevertheless, the problem may be improved by increasing the regeneration temperature or by direct thermal regeneration at higher temperature which is favorable for the decomposition of carbamate^22^,^23^.

Compared to our previously adopted tributylamine + n-butnol system, the diisobutylamine + n-octanol system showed many advantages such as low phase ratio, better temperature adaptation, better regeneration performance, and, in particular, excellent enhancement performance for Mg^2+^ precipitation. It can be conclusively summed up that the fixation of CO_2_ through enhancing carbonation in Ca^2+^ /Mg^2+^-rich aqueous solutions is feasible and effective by using the novel diisobutylamine + n-octanol system as an enhancement medium.

In sum, an overall profile of CO_2_ fixation was confirmed and optimized here through enhancing the carbonation reactionsin Ca^2+^/Mg^2+^-rich aqueous solutions. The diisobutylamine + n-octanol system was used as a novel carbonation enhancement agent based on a screening via apparent basicity measurement of amine-diluent system. The series of experiments carried out in a self-designed experimental setup at room temperatures indicated: for an artificial solution containing about twice concentrations of Ca^2+^ and Mg^2+^ of those in natural seawater, 98% Ca^2+^ and 91% Mg^2+^ could be converted to carbonate precipitation within 5 min of reaction with the bubbling-in of CO_2_; the precipitate products took the form of calcium and magnesium carbonates; both the phase ratio and the temperature had influences on the carbonation process; the organic amine system could be regenerated by using carbide slag as the regeneration agent and still showed attractive enhancement performances after 7 rounds of carbonation-regeneration experiments.

The CO_2_ fixation conception built up here has some advantages over the routine CCS routes and may have good application potentials. 1) The CO_2_ capture and sequestration can be realized within one single process and there is no need for the CO_2_ separation, liquification and transportation. The transformation from gaseous CO_2_ to solid carbonates can be completed at room temperatures and within a period as short as several minutes. 2) The precipitate products are stable calcium and magnesium carbonates, which have vast application potentials in industrial fields owing to their fine particle sizes, white color appearance and chemical composition stability. 3) The Ca^2+^/Mg^2+^-rich aqueous solutions are widely distributed and abundant in both categories and quantities, as provides good implementation possibilities for this novel conception. For instance, concentrated seawater or brine effluents, which are usually discharged as waste in desalination industry, can extend their value chains through CO_2_ fixation; meanwhile, the removal of Ca^2+^ and Mg^2+^ from these solutions may trigger lower scaling and lead to higher ratios of fresh water production.

Further work is just being focused on another method of regeneration for the insoluble amine, where calcium and/or magnesium silicates are tested as the regeneration agent. If it works well, a novel quick CO_2_ mineralization process may also be built up by enhancing the dissolution of silicate minerals and the carbonation of CO_2_^24^.

## Additional Information

**How to cite this article**: Wang, W. *et al*. Experiment and Optimization for Simultaneous Carbonation of Ca^2+^ and Mg^2+^ in A Two-phase System of Insoluble Diisobutylamine and Aqueous Solution. *Sci. Rep*. **5**, 10862; doi: 10.1038/srep10862 (2015).
